# Specific detection of *Staphylococcus aureus* infection and marker for Alzheimer disease by surface enhanced Raman spectroscopy using silver and gold nanoparticle-coated magnetic polystyrene beads

**DOI:** 10.1038/s41598-021-84793-7

**Published:** 2021-03-18

**Authors:** Robert Prucek, Aleš Panáček, Žaneta Gajdová, Renata Večeřová, Libor Kvítek, Jiří Gallo, Milan Kolář

**Affiliations:** 1 Department of Physical Chemistry, Faculty of Science, Palacký University Olomouc, 17 Listopadu 12, 771 46 Olomouc, Czech Republic; 2 Department of Microbiology, Faculty of Medicine and Dentistry, Palacký University Olomouc, Hněvotínská 3, 775 15 Olomouc, Czech Republic; 3 Department of Orthopaedics, Faculty of Medicine and Dentistry, Palacký University Olomouc, I. P. Pavlova 6, 77520 Olomouc, Czech Republic

**Keywords:** Microbiology, Biomarkers, Chemistry, Materials science, Nanoscience and technology

## Abstract

Targeted and effective therapy of diseases demands utilization of rapid methods of identification of the given markers. Surface enhanced Raman spectroscopy (SERS) in conjunction with streptavidin–biotin complex is a promising alternative to culture or PCR based methods used for such purposes. Many biotinylated antibodies are available on the market and so this system offers a powerful tool for many analytical applications. Here, we present a very fast and easy-to-use procedure for preparation of streptavidin coated magnetic polystyrene–Au (or Ag) nanocomposite particles as efficient substrate for surface SERS purposes. As a precursor for the preparation of SERS active and magnetically separable composite, commercially available streptavidin coated polystyrene (PS) microparticles with a magnetic core were utilized. These composites of PS particles with silver or gold nanoparticles were prepared by reducing Au(III) or Ag(I) ions using ascorbic acid or dopamine. The choice of the reducing agent influences the morphology and the size of the prepared Ag or Au particles (15–100 nm). The prepare composites were also characterized by HR-TEM images, mapping of elements and also magnetization measurements. The content of Au and Ag was determined by AAS analysis. The synthesized composites have a significantly lower density against magnetic composites based on iron oxides, which considerably decreases the tendency to sedimentation. The polystyrene shell on a magnetic iron oxide core also pronouncedly reduces the inclination to particle aggregation. Moreover, the preparation and purification of this SERS substrate takes only a few minutes. The PS composite with thorny Au particles with the size of approximately 100 nm prepared was utilized for specific and selective detection of *Staphylococcus aureus* infection in joint knee fluid (PJI) and tau protein (marker for Alzheimer disease).

## Introduction

Specific and effective therapy of diseases highly relies on utilization of rapid identification methods of the given disease marker. Ideal methods for rapid specific identification would include those that require minimal sample volume and allow rapid sample detection and characterization. Surface enhanced Raman spectroscopy (SERS) seems to be one of the prospective methods; recently, it has become an increasingly relevant analytical method that enables to detect molecules in the area of pico to femto molar concentrations^1^. The most widely used materials for SERS include silver or gold; such substrates based on the mentioned coin metals have been exploited for detection or determination of many important or dangerous molecules such as bisphenol A^2^, polycyclic aromatic hydrocarbons^3^, antibiotics^4^, bacteria or yeast^5, 6^, and bacteria markers^7^.

It has been shown recently that anisotropic nanoparticles with sharp tips on the surface can promote extraordinary enhancement in the Raman signal in comparison with spherical or smoother nanoparticles^8, 9^, e.g. thorny Au nanoparticles^10–12^ or Au nanostars^8, 9, 13^. Such structures include lots of “hot spots” enabling very high enhancement of the electromagnetic field around the particle. Gold nanostars are biocompatible nanostructures that represent a promising nanoplatform for various biomedical applications such as photodynamic therapy^14^, photothermal therapy^15^, theranostics^16, 17^, biosensing^18^, and as effective SERS substrates^19–23^ because of the above-mentioned reason.

Nanoparticles enable fast interaction with analytes to shorten the detection time. Nevertheless, the direct application of colloids in SERS measurements can be limited because of the variation of the SERS performance caused by eventual aggregation of the colloids. Improvement of the practical utilization can be reached by deposition of nanoparticles on a solid substrate^24^. Another possible way to increase efficiency and/or applicability is to synthesize composites of silver or gold nanoparticles on an appropriate support. For example, composites of silver nanoparticles and reduced graphene oxide have been exploited for detection of 1-pyrenecarboxylic acid^25^ or for bacteria detection^26^. Multifunctional hybrid structures have been prepared to integrate the concentration ability of magnetic particles with the surface plasmonic effect of metal colloid particles^27–29^. The hybrid material combines both the SERS enhancement by the noble metal nanostructures and the ease in recovery of the particles from the reaction mixture using an external magnet^30–32^. The advantage seems to lie in the combination of magnetic substrates and silver or gold particles. Such composites of silver or gold nanoparticles with a magnetic component have been exploited for detection of various analytes such as cyanides, hydrogen peroxide, and nitrites^27^, or for detection of methotrexate^33^, genetically modified organisms^34^, or antibiotics^35^.

A number of publications related to detection of the above-mentioned analytes exploit non-specific interaction of detected compounds and silver or gold particles or their layers, which serve as Raman signal enhancers. A promising way to the selective detection of the given molecules is offered by streptavidin–biotin or streptavidin–biotinylated antibody systems, which represents one of the strongest known non-covalent interactions with an association constant of 10^15^ M^−1^. The protein is resistant to a number of extreme conditions such as temperature, denaturants, and pH values, but biotin exhibits nonspecific binding of avidin. As an alternative, the analogous protein streptavidin (SA) isolated from the bacteria *Streptomyces avidinii* shows a similar affinity to avidin^28, 36^. The applicability of the streptavidin–biotin complex^37–39^ or other similar key-lock systems^40^ can be highly enhanced by anchoring a given antibody on magnetic carriers enabling selective capturing of required antigen and its eventual separation, purification, and pre-concentration for subsequent analysis. For example, the detection and separation of pathoghens^41^, multiple viral antigens^40, 42^, bacterial DNA^43^, melamine^44^, or immunoglobulin IgG^38, 45^ were already reported using such techniques. However, many of SERS immunoassay based procedures have been mostly indirect and required Raman reporter molecules to provide an intensive Raman signal^29, 40, 42, 44^. The approach published by Balzerova et al., which is based on a label free SERS method, seems to be more advantageous. In this case, the prepared Fe_3_O_4_@Ag composite is subsequently modified by carboxy polyethylene glycol and carboxymethylchitosan with further immobilization of streptavidin. However, this procedure involves five reaction steps within several hours and requires careful washing between individual stages, which can be seen as a potential drawback of this approach resulted from the length of the synthesis and possible irreproducibility of the preparation of the mentioned composite. A similar several-step procedure has been used for synthesis of core–shell composite Fe_3_O_4_@SiO_2_@Au modified by streptavidin^46^. However, in this case, the separation of the magnetic particles covered by a given antibody–antigen complex was performed at first and then, the modified gold colloidal particles serving as Raman signal enhancers were added to the reaction mixture.

Here, we offer a very fast and easy-to-use procedure for preparation of streptavidin coated magnetic polystyrene gold or silver particles composites as highly efficient SERS substrate. The presented composite represents a biosensor substrate with a high variability of use for various applications and also enables reducing the sample pre-treatment, time, and cost of the analysis. The proposed strategy holds a great potential for highly sensitive and selective analysis of target markers in complex biological samples.

## Results and discussion

A well-known streptavidin–biotin complex offers an interesting method for selective separation and/or identification of biologically important molecules. As mentioned above, many biotinylated antibodies are available on the market or biotinylation of selected antibodies could be performed in laboratories; this system thus represents a powerful tool for many analytical applications^36, 48^. As a precursor for the preparation of the SERS active and magnetically separable composite, commercially available streptavidin coated polystyrene microparticles with a magnetic core were utilized. These composites of polystyrene particles with silver or gold nanoparticles were prepared by reducing the Au(III) ions or Ag(I) ions with ascorbic acid and dopamine, which are vital for human body, and they serve as a powerful antioxidant and a neurotransmitter in brain, respectively.

**Figure 1:**
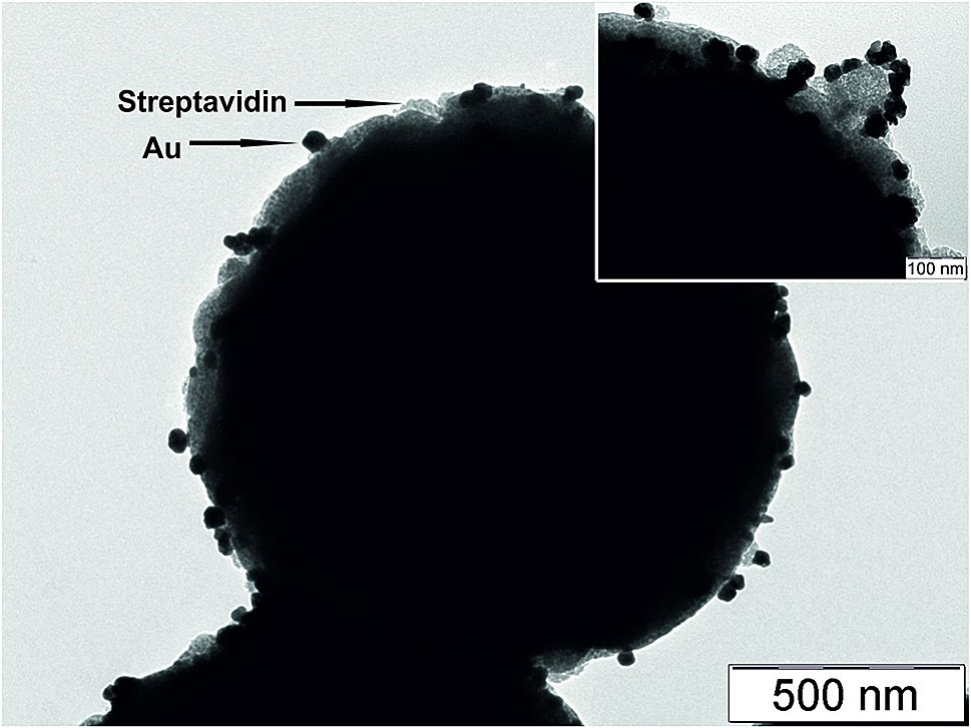
TEM images of Strep@mPS-AuNPs composite prepared using ascorbic acid as a reducing agent for gold(III) ions.

**Figure 2:**
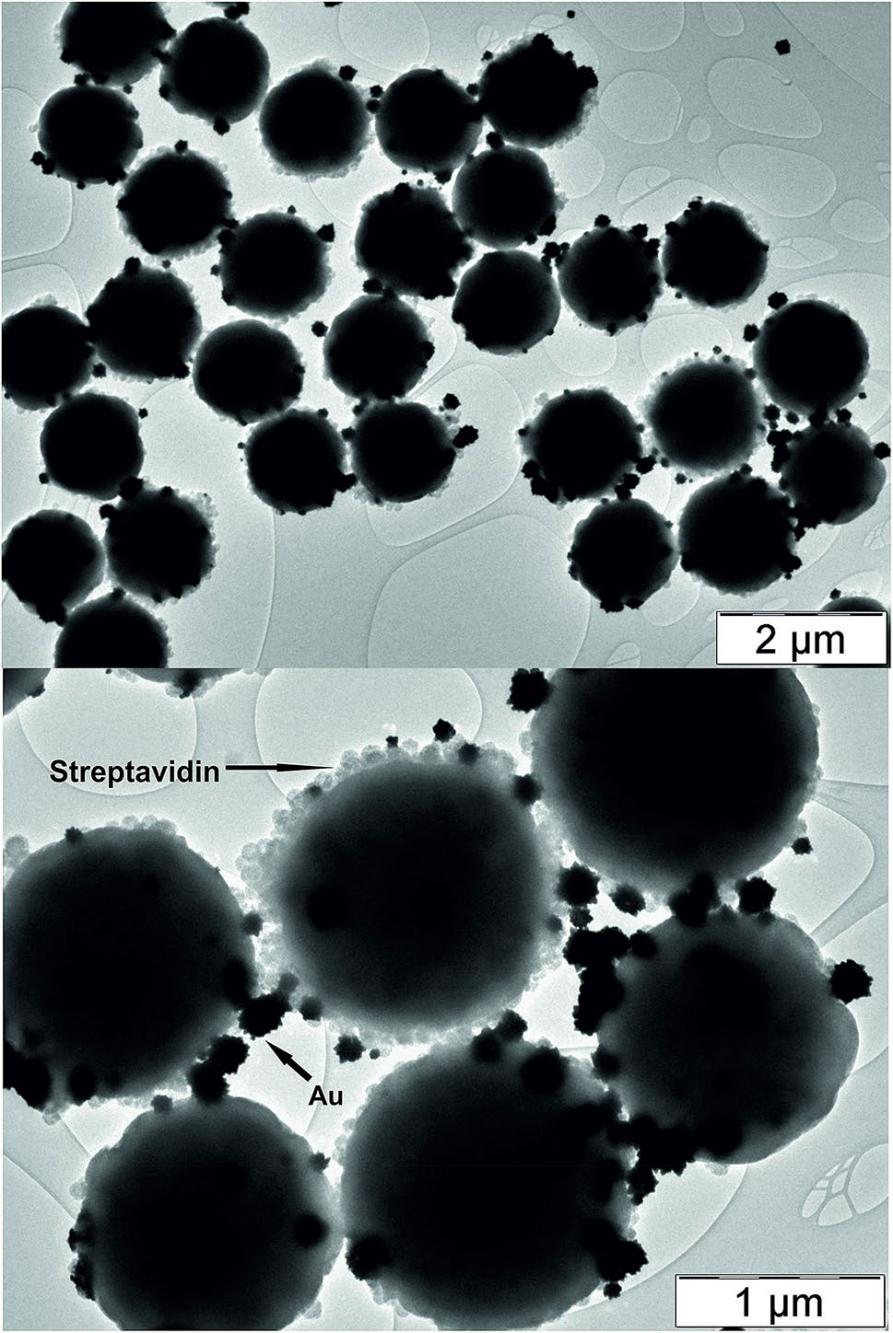
TEM images of Strep@mPS-tAuNPs composite prepared using dopamine as a reducing agent for gold(III) ions.

Gold ions can be reduced even in acidic environment using the mentioned reducing agents. The redox potential of Au(III) ions is approx. + 1.50 V in the acidic environment and the redox potential at pH 3 of ascorbic acid and dopamine is + 0.24 V and + 0.61 V, respectively. The difference between redox potentials is therefore sufficient for the reduction of the gold ions and for the subsequent formation of gold particles. Figures 1 and 2 show TEM images of Strep@mPS-AuNPs composite (Au/magnetic streptavidin coated polystyrene particles composite) synthesized through reduction of gold ions using ascorbic acid and dopamine. In the case of the ascorbic acid, the average particle size of gold was 35 nm (Fig. 1), whereas when dopamine was used as the reducing substance, the average particle size of Au grew up to 100 nm. Moreover, the morphology of the particles changed substantially and the thorny Au particles were observed under these experimental conditions (Fig. 2).

This Strep@mPS-AuNPs composite with thorny morphology of Au particles subsequently proved to be the most effective substrate for the surface enhanced Raman spectroscopy experiments (see below).

**Figure 3:**
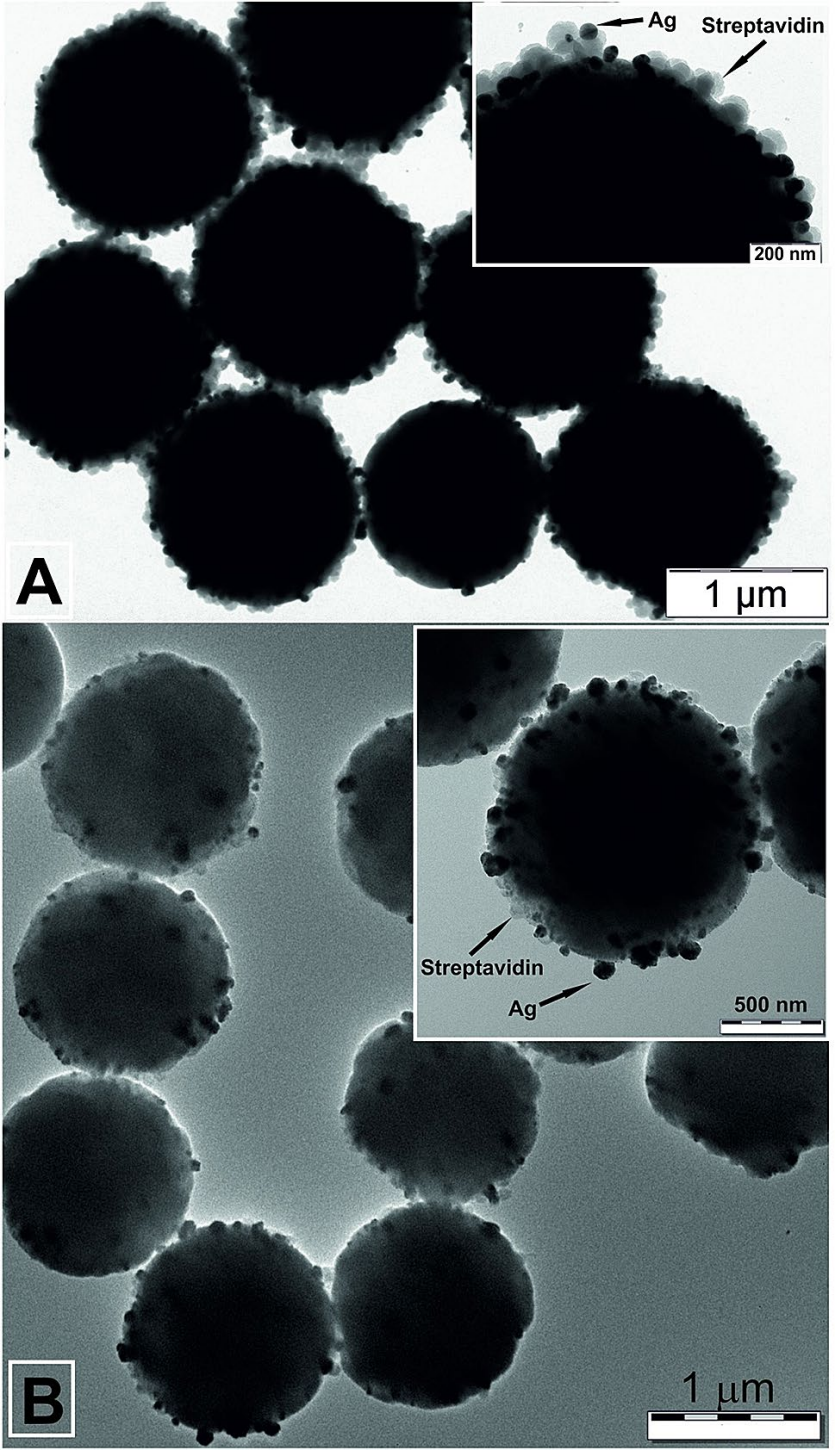
TEM images of Strep@mPS-AgNPs composite prepared using ascorbic acid (**A**) and dopamine (**B**) as a reducing agent for silver (I) ions.

The reduction of silver ions by the given reducing agents is not feasible in the acidic environment. In this case, it is necessary to decrease the redox potential of ascorbic acid and dopamine by increasing the pH value of the reaction mixture. At a pH of about 10.5, the redox potential of ascorbic acid and dopamine is + 0.08 V and + 0.22 V, respectively. However, the formation of silver oxide precipitate occurs at this pH value. To keep the silver ions in a homogeneous state at alkaline pH values, it is necessary to exploit a complex agent such as sulfite or ammonia^49–51^. Bonding silver ions to the complex compound reduces the redox potential from + 0.80 to + 0.58 V in the case of ammonia with a final concentration equal to 1.25 mmol dm^−3^. The difference between redox potential of silver ions and the reducing agent is sufficient for reduction of silver ions. Figure 3 shows TEM images of Strep@mPS-AgNPs composite (Ag/magnetic streptavidin coated polystyrene particles composite) prepared by reduction with ascorbic acid and dopamine, respectively. The average particle sizes were 40 nm (ascorbic acid) and 70 nm (dopamine).

These observations of average Au and Ag particle size dependences are consistent with the fact that the average particle size of the synthesized Au and Ag particles can be varied by changing the difference between the redox potential of the reducing agents and the ionic metal species (free or in a complex form) of the given metal. However, the dimensions of the prepared metal particles may not be affected only by this parameter. The structure and acid base properties of the reducing substance also play a substantial role in the formation process of metal particles^50^. Silver and especially gold ions or particles have a strong affinity to nitrogen atoms, which represent another factor that can influence the formation of particles especially in the case of the gold (thorny Au particles, Fig. 2). Moreover, dopamine contains an aromatic core in its structure, which is capable of interacting with the surface of the polystyrene particles and can represent a linker between the magnetic PS particles and the formed Ag or Au particles.

The presented magnetic PS and Ag or Au particle composites have a significantly lower density against magnetic composites based on iron oxides which considerably decrease tendency to sedimentation. Also, the polystyrene shell on a magnetic iron oxide core pronouncedly reduces the propensity to aggregation because of weaker magnetic forces. Both of these factors contribute to an increase in the reproducibility of the SERS measurement.

**Figure 4:**
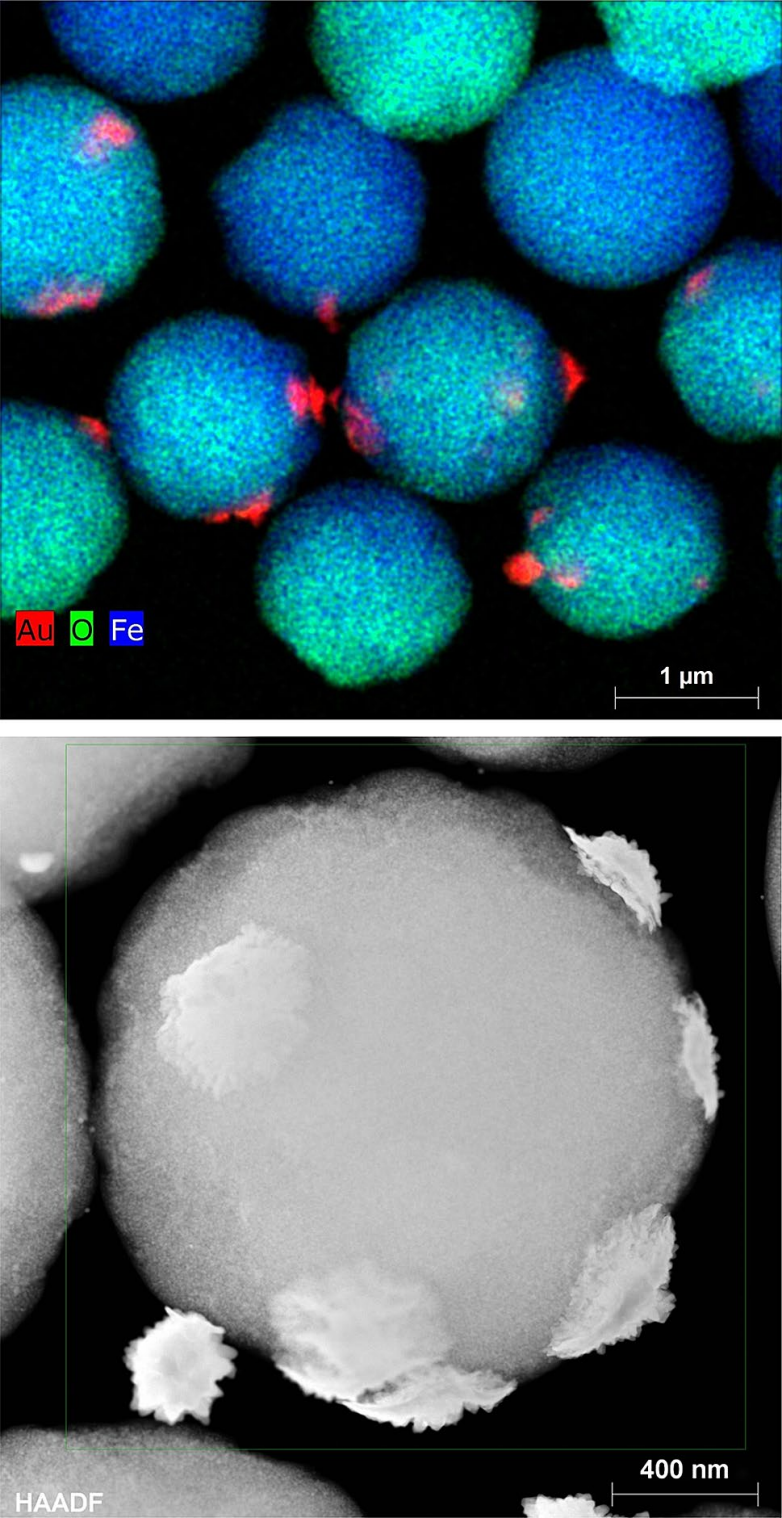
HR-TEM images and mapping of elements (Au, Fe) presence. Other images are involved in “Supporting information” (Fig. S2).

The presence of gold and silver were confirmed by AAS measurements and also by HR-TEM mapping of elementents, for example for gold thorny particles as showed in Fig. 4. Magnetic characteristic of polystyrene particles with magnetic core supplied from Sigma-Aldrich (Merck) were verified through SQUID measurement (see Fig. S1 in “Supporting Information”).

**Figure 5:**
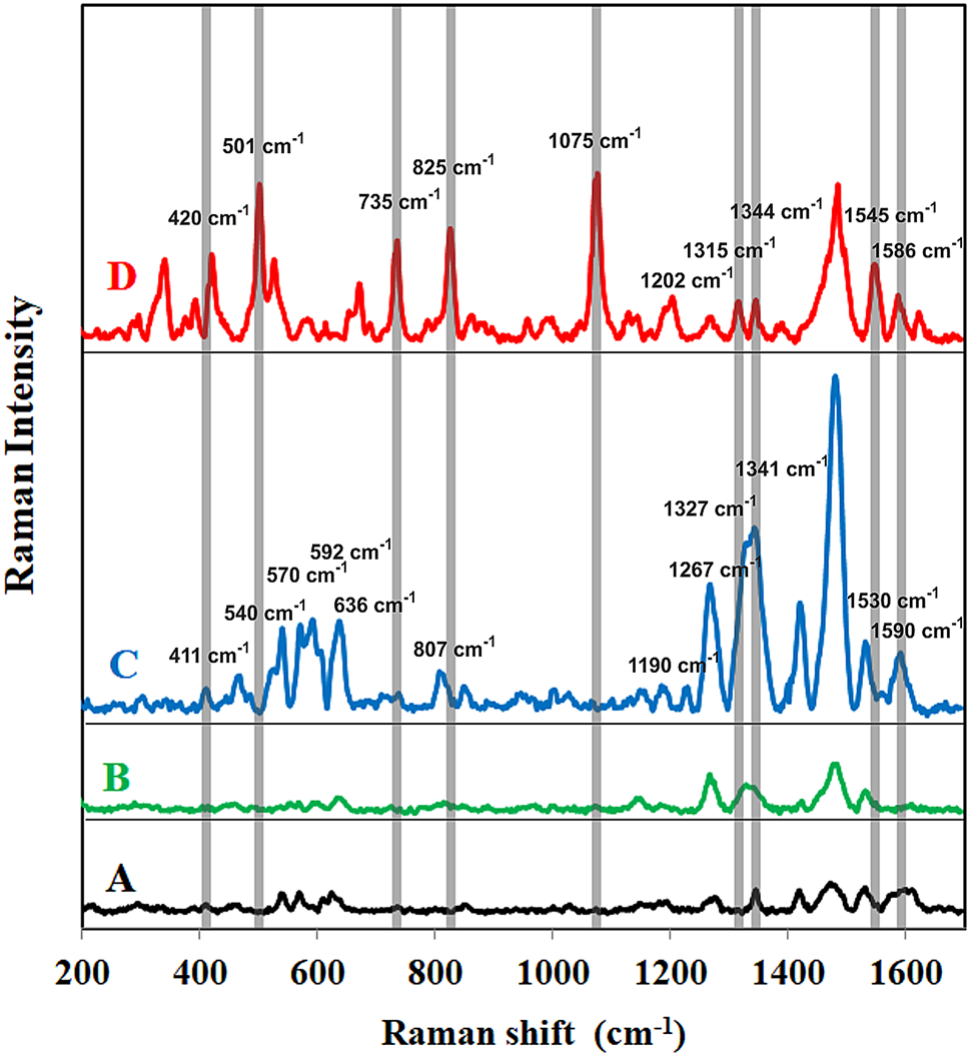
(**A**) Surface enhanced Raman (SER) spectrum of streptavidin coated polystyrene particles covered by thorny Au particles (Strep@PS-tAuNPs), (**B**) SER spectrum of Strep@PS-tAuNPs after the addition of biotinylated antibody to Protein A (antiP@Strep@mPS-tAuNPs, (**C**) SER spectrum of antiP@Strep@mPS-tAuNPs after the addition of Protein A, (**D**) SER spectrum of Protein A@antiP@Strep@mPS-tAuNPs after the addition of real sterile joint knee fluid sample obtained by puncture from patients with osteoarthritis spiked subsequently by *Staphylococcus aureus* (STAU/JKF@antiP@Strep@mPS-tAuNPs). Laser excitation wavelength was 785 nm.

As mentioned above, the PS composites with thorny Au particles prepared via reduction using dopamine proved to be the most efficient substrate for the SERS measurement purposes. Therefore, this Strep@mPS-tAuNPs composite was tested as a possibly effective SERS substrate for detection of protein A, which is a marker of *Staphylococcus aureus* (in model sample and in a real joint knee fluid sample) and for the detection of Tau protein. Current estimates suggest that prosthetic joint infection complications occur in up to 3% of primary hip and knee arthroplasties and up to 15.4% and 25% of revision hip and knee arthroplasties, respectively^52^. The pathogenesis of PJI is related to the presence of biofilm-forming microorganisms, the most commonly Staphylococci^53^. Figure 5 (spectrum A) shows surface enhanced Raman (SER) spectrum of Strep@mPS-tAuNPs, where weak bands can be seen in the regions of 500–650 cm^−1^ and 1300–1600 cm^−1^, which can be ascribed to streptavidin being bounded to the surface of polystyrene beads. After the addition of biotinylated antibody to Protein A to the Strep@mPS-tAuNPs, the intensity of some bands in the region 500–650 cm^−1^ decrease. On the other hand, the intensity of bands in the region between 1300 and 1600 cm^−1^ increased (antiP@Strep@mPS-tAuNPs, spectrum B). After subsequent addition of Protein A to antiP@Strep@mPS-tAuNPs (ProteinA@antiP@Strep@mPS-tAuNPs, spectrum C), new bands located at 540, 570, 591, and 636 cm^−1^ and at 1148, 1190, 1228, 1327, 1341, 1419, and 1590 cm^−1^ emerged. The first group of bands can be attributed to skeletal δ (CC_3_) and δ (CH) deformations of aminoacids such as alanine, valine, glutamate, tyrosine, tryptophan, histidine, proline present in the structure of Protein A. The second group of bands most likely originate from alanine (1479 cm^−1^), valine (1341 cm^−1^), and phenylalanine (1590 cm^−1^). The band at 1228 cm^−1^ can be attributed to ν (CN) stretching mode, the band at 1419 cm^−1^ corresponds to the carboxylate vibrational mode (COO^−^ stretch), and the bands around 1330 cm^−1^ and 1550 cm^−1^ can be ascribed to C–H bend, to amide III mode, and amide II mode of aminoacids, respectively^54^.

However, new bands also emerged example at 501, 735, and 1075 cm^−1^, which most probably corresponds to a glycosidic ring mode of the peptidoglycan cell wall building blocks, poly-*N*-acetylglucoseamine and *N*-acetylmuramic acid^54^.

**Figure 6:**
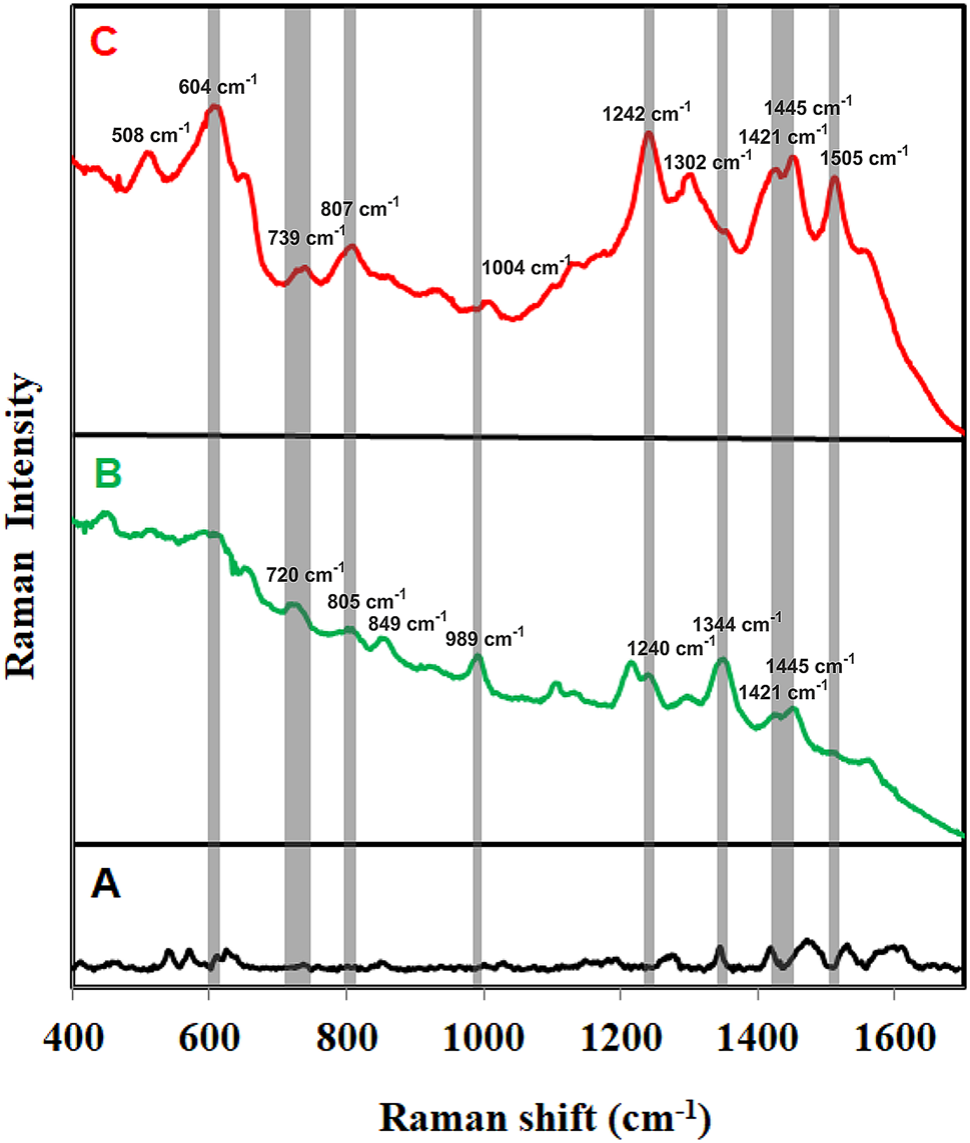
(**A**) Surface enhanced Raman (SER) spectrum of streptavidin coated polystyrene particles covered by thorny Au particles (Strep@PS-tAuNPs), (**B**) SER spectrum of Strep@PS-tAuNPs after the addition of biotinylated antibody to tau protein (anti-tau@Strep@mPS-tAuNPs), (**C**) SER spectrum of anti-tau@Strep@mPS-tAuNPs after the addition of tau protein (tau@anti-tau@Strep@mPS-tAuNPs). Laser excitation wavelength was 785 nm.

Tau (tubulin-associated unit) is expressed in neurons and constitutes the neuronal microtubules network. Microtubules serve as tracks for axonal transport. Tau proteins also establish some links between microtubules and other cytoskeletal elements or proteins. Six tau protein isoforms exist in human brain tissue. Aberrant assembly of Tau proteins into aggregates is accompanied by synaptic dysfunction and neural cell death leading to Alzheimer’s disease. Increased concentrations of cerebrospinal fluid total Tau and phosphorylated tau, as well as decreased amyloid β 42 peptide, are biomarkers for Alzheimer’s disease (AD. It is estimated that more than 130 million people worldwide live with dementia in a year 2050^55, 56^. Normal physiological concentration of Tau protein is 244 ± 156 ng L^−1^. In the case of the Alzheimer’s disease, the pathological value of Tau protein in cerebrospinal fluid is 587 ± 365 ng L^−1^^57^. Figure 6 (spectrum A) shows surface enhanced Raman (SER) spectrum of Strep@mPS-tAuNPs. After addition of biotinylated antibody to tau protein to the Strep@mPS-tAuNPs (anti-tau@Strep@mPS-tAuNPs, spectrum B), new bands located at 720, 805, 849, 983, 1240, 1344, 1421 and 1590 cm^−1^ emerged. These bands can be ascribed to skeletal δ (CC_3_) and δ (CH) deformations, ν (CN) stretching mode, carboxylate vibrational mode (COO^−^ stretch), and to amide III mode and amide II mode of aminoacids, respectively as mentioned in the previous example. After subsequent addition of tau protein to anti-tau@Strep@mPS-tAuNPs (tau@anti-tau@Strep@mPS-tAuNPs, spectrum C), some bands were shifted (720–739 cm^−1^, 989–1004 cm^−1^) and some new bands at 508, 1302, and 1505 cm^−1^ appeared, which indicates bonding of tau protein to anti-tau biotinylated antibody.

## Conclusion

The presented approach represents simple, rapid, and easy-to-use synthetic procedure of efficient substrate for selective separation and consequent identification using surface enhanced Raman spectroscopy. As a precursor for the preparation of such magnetically separable composites, a commercially available streptavidin coated polystyrene microparticles with a magnetic core were utilized as a magnetic and separation enabling part. The surface of these particles was covered by Ag or Au particles with different size and morphology through the reduction of silver and gold ions using ascorbic acid or dopamine. The prepared composites have a significantly lower density and tendency to aggregation against magnetic composites based on iron oxides, which increases the reproducibility of the SERS measurement. As many biotinylated antibodies are available on the market, the presented composite represents a biosensor substrate with a wide range of use for various applications and it also enables reducing the sample pre-treatment, time, and cost of the analysis. The PS composites with thorny Au particles prepared via reduction using dopamine proved to be the most efficient substrate for the SERS measurement purposes and this substrate was utilized and tested for specific and selective detection of protein A, which is a marker of *Staphylococcus aureus* (in the model sample and in a real joint knee fluid sample) and for the detection of Tau protein. The proposed strategy holds great potential for highly sensitive and selective analysis of markers in complex biological samples.

## Materials and chemicals

Polystyrene particles with magnetic core and streptavidin coated (Sigma-Aldrich, particle size 1 µm ± 0.1 µm, binding capacity for FITC-Biotin > 600 pmol mg^−1^, iron oxide content ≥ 20%), gold chloride trihydrate (≥ 99.9%), silver nitrate (ACS reagent ≥ 99.9%), dopamine hydrochloride, ascorbic acid (reagent grade), sodium hydroxide (p.a.), ammonium hydroxide solution (ACS reagent), phosphate buffered saline (10 × concentrate, BioPerformance Certified), (+)-Biotin N-hydroxysuccinimide ester (≥ 98%), monoclonal Anti-Protein A-Biotin antibody produced in mouse (B3150), Protein A from *Staphylococcus aureus* (P7837), Anti-TAU antibody produced in rabbit (SAB4501821), Tau Protein Ladder, 6 isoforms human (T7951). All chemicals were purchased from Sigma-Aldrich. Deionized water was used for all experiments with conductivity 0.05 µS cm^−1^ obtained from instrument Aqual 29 (Mersi).

### Instrumentation

Particle size of samples were performed by transmission electron microscopy (TEM) on a JEOL JEM-2010 transmission electron microscope equipped with a LaB6 cathode (accelerating voltage of 160 kV). Raman spectra were recorded using the portable Raman spectrometer i-Raman Plus (BW Tek), 785 nm excitation wavelength. The SERS spectra were acquired in the range from 400 to 1800 cm^−1^, 10 s scan time and six accumulations were used. The laser light incident onto a sample was 50 mW. All the spectra were measured at room temperature. Gold and silver concentrations were determined by the AAS-ETA technique with graphite furnace using a ContrAA 600 (Analytik Jena AG, Germany) equipped with a high-resolution Echelle double monochromator (spectral band width of 2 pm at 200 nm) and with a continuum radiation source (xenon lamp). The absorption lines used for Ag and Au concentration analyses were 328.068 nm and 242.795 nm, respectively.

### Synthesis of Au/magnetic or Ag/magnetic streptavidin coated polystyrene particles composite (Strep@mPS-AuNPs)

**Figure 7:**
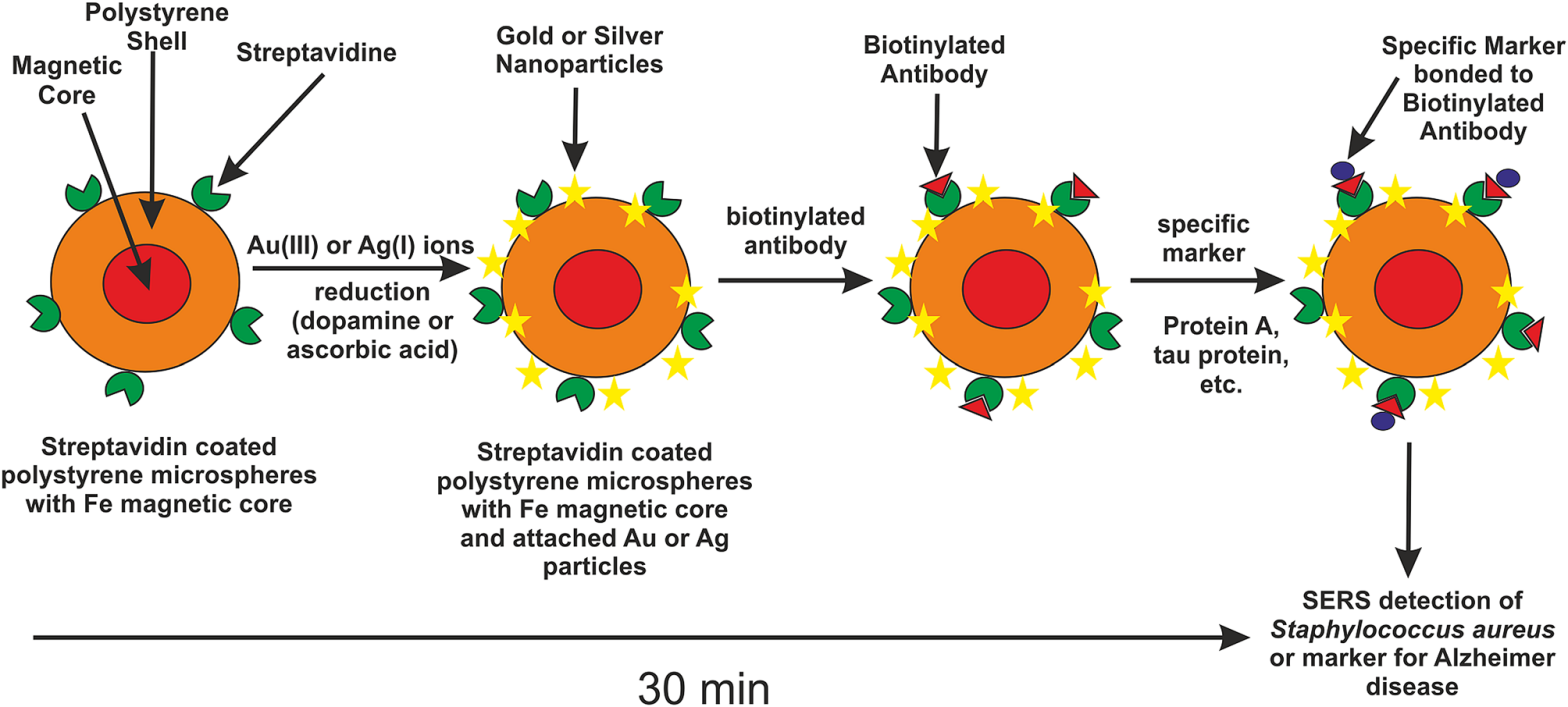
Scheme showing the individual steps in the preparation of the magnetic SERS substrate for specific detection.

The amount of 0.2 mL (1% w/v) of commercially available polystyrene particles (1 µm in size) with a magnetic core and being covered by streptavidin was mixed with 8.3 mL of distilled water. After that, 0.5 mL of gold chloride solution or silver nitrate (5∙10^−3^ mol dm^−3^) was added and the reaction mixture was stirred for 3 min at 500 rpm. Finally, 1 mL solution (10^−2^ mol dm^−3^) of dopamine or ascorbic acid was added and the reaction solution was stirred for the next 3 min. The final concentrations of Au or Ag and the reducing agents were 2.5∙10^−4^ mol dm^−3^ and 10^−3^ mol dm^−3^, respectively. The final pH value was equal to 2.8 for Au compositeand 10.5 for Ag composite. The schema of preparation of composite is depicted in Fig. 7.

### Preparation of bacterial samples

Testing samples were prepared using the bacterial species *Staphylococcus aureus* CCM 3953 (STAU). Well-isolated colonies were transferred into 5 mL of Mueller–Hinton broth so that the resulting turbidity was equivalent to a McFarland standard of 1.0 (approximate cell density of 3 × 10^8^ CFU mL^−1^). The inoculum density was determined by measuring the samples’ optical density with a densitometer (Densi-La-Meter; LACHEMA, Czech Republic), and the bacterial suspensions were then cultured at 37 °C for 24 h. After incubation, the broth was centrifuged at 2000 rpm for 10 min. The supernatant was then removed, and 10 mL of distilled water was added to the pellet^58, 59^.

### Preparation of real-matrix samples

The real-matrix sample was prepared from sterile knee joint fluid obtained by puncture from a patient with osteoarthritis. To verify the sterility of it, the initial joint fluid was inoculated onto blood agar (Trios, Ltd.) and into Mueller–Hinton broth (HiMedia) and cultivated. Real-matrix samples for testing were then prepared by spiking samples of the joint fluid in a 1:1 ratio with bacterial lysate. All joint fluid samples were obtained under standard conditions with a written informed patient consent, and the study was approved by the local Ethics Committee. The University Hospital Ethical Committee in Olomouc, Czech Republic approved the study employing joint fluid samples in accordance with the Helsinki Declaration. In order to obtain a sample of aseptic synovial joint fluid an appropriate patient with symptomatic knee osteoarthritis was identified. A knee puncture was a part of therapeutic guideline used in osteoarthritic patients with knee effusion at our University Hospital clinic. An informed written consent was obtained before the puncture of all patients^39^.

### Samples for SERS measurement

Samples of Strep@mPS-AuNPs or Strep@mPS-AgNPs were magnetically separated, the supernatant was removed and distilled water was added to the separated particles with the same volume of samples as in the initial state. This procedure was repeated three times. Then, the samples were used for purposes of Raman spectra measurements. At first, Raman spectra of Strep@mPS-AuNPs or Strep@mPS-AgNPs were collected. The Strep@mPS-AuNPs composite with thorny morphology of Au particles proved to be the most effective substrate for the surface enhanced Raman spectroscopy experiments (Strep@mPS-tAuNPs) and it utilized for all the other experiments.

With regard to measuring *Staphylococcus aureus* in the sterile joint knee fluid, the next steps were measurement of the following samples: (a) Strep@mPS-tAuNPs with biotinylated antibody to Protein A (antiP@Strep@mPS-tAuNPs); (b) Strep@mPS-tAuNPs with biotinylated antibody to Protein A and subsequent addition of *Protein A* (Protein A@antiP@Strep@mPS-tAuNPs; (c) Strep@mPS-tAuNPs with biotinylated antibody to Protein A and subsequent addition of a sterile joint knee fluid (JKF) sample spiked by *Staphylococcus aureus* bacteria (STAU/JKF@antiP@Strep@mPS-tAuNPs). To prepare the Strep@mPS-tAuNPs sample with biotinylated antibody to Protein A, 50 μL solution of Strep@mPS-tAuNPs was mixed with 10 μL solution of biotinylated antibody to Protein A and the mixture was shaken for 3 min. To prepare the Strep@mPS-tAuNPs sample with bounded biotinylated antibody to Protein A and Protein A, 50 μL solution of Strep@mPS-tAuNPs with bounded biotinylated antibody to Protein A was mixed with 10 μL solution of Protein A and the mixture was shaken for 3 min. Prior to the analysis of real-matrix samples of joint fluid spiked with live bacterial cells, the samples were diluted tenfold with deionized water to modify their consistency. The real-matrix sample of knee joint fluid spiked with live bacterial cells was then analyzed using the following SERS protocol. A sample volume of 50 μL was mixed with 50 μL solution of the antiP@Strep@mPS-tAuNPs, and the mixture was shaken for 3 min. In all cases, a 10 μL aliquot of the solution was then dropped onto a glass slide for the purposes of SERS measurement.

As for measuring Tau protein, the next steps involved measurement of the following samples: (a) Strep@mPS-tAuNPs with biotinylated antibody to tau protein (anti-Tau@Strep@mPS-tAuNPs); (b) anti-tau@Strep@mPS-tAuNPs and Tau protein (Tau@anti-tau@Strep@mPS-tAuNPs). The anti tau protein was biotinylated using biotin N-hydroxysuccinimid^47^. The samples were prepared by mixing 20 μL of Strep@mPS-tAuNPs, 2 μL of biotinylated antibody to tau protein solution (100 times diluted using PBS buffer from the stock solution) and 2 μL of tau protein solution (100 times diluted using PBS buffer from the stock solution). In the case of sample (a) and (b), the volume was adjusted to 30 μL using PBS buffer. The final concentration of Tau protein in the sample was 25 ng mL^−1^. In all cases, a 10 μL aliquot of the solution was then dropped onto a glass slide for the purposes of SERS measurement. The mixtures were shaken for 3 min between the steps.

The overall preparation process of the magnetic SERS substrate for specific detection is illustrated in Fig. 7.

## Supplementary Information

Supplementary Information.
