# Supplementary material for: Specific detection of Staphylococcus aureus infection and marker for Alzheimer disease by surface enhanced Raman spectroscopy using silver and gold nanoparticle-coated magnetic polystyrene beads
Source: Sci Rep. 2021 Mar 18;11:6240. doi: 10.1038/s41598-021-84793-7 (PMC7973519; doi:10.1038/s41598-021-84793-7)
Supplement: Supplementary file 1 — Supplementary Information. [file 41598_2021_84793_MOESM1_ESM.docx]

**Specific detection of *Staphylococcus aureus* infection and marker for Alzheimer disease by surface enhanced Raman spectroscopy using silver and gold nanoparticle-coated magnetic polystyrene beads**

Robert Prucek^1^*, Aleš Panáček^1^, Žaneta Gajdová^1^, Renata Večeřová^2^, Libor Kvítek^1^, Jiří Gallo^3^, and Milan Kolář^2^

^1^ Department of Physical Chemistry, Faculty of Science, Palacký University Olomouc, 17 Listopadu 12, 771 46 Olomouc, Czech Republic.

^2^ Department of Microbiology, Faculty of Medicine and Dentistry, Palacký University Olomouc, Hněvotínská 3, 775 15 Olomouc,Czech Republic

^3^ Department of Orthopaedics, Faculty of Medicine and Dentistry, Palacký University Olomouc, I. P. Pavlova 6, 77520 Olomouc, Czech Republic

^*^ Corresponding author: Robert Prucek, robert.prucek@upol.cz


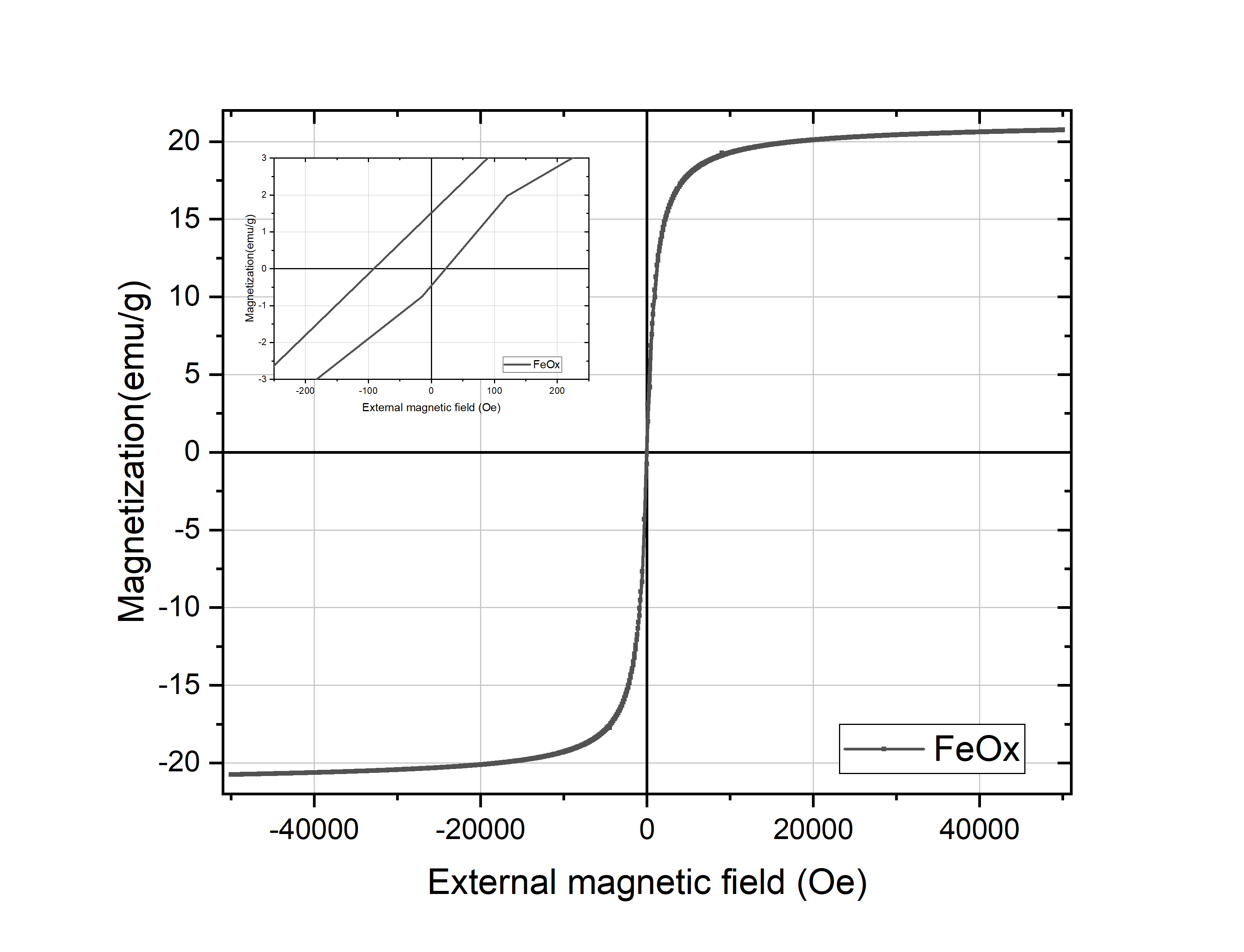


**Figure S1:** Magnetic measurements of Strep@mPS-tAuNPs composite prepared using dopamine as a reducing agent for gold(III) ions.

| H_C1_ | 22.8 Oe | M_R1_ | 1.5 emu/g | M_S1_ | 20.73 emu/g |
| --- | --- | --- | --- | --- | --- |
| H_C2_ | -91.6 Oe | M_R2_ | -0.5 emu/g | M_S2_ | 20.75 emu/g |
| H_C_ | 57.2 Oe | M_R_ | 1.0 emu/g | M_S_ | 20.74 emu/g |

Sample was analyzed using a Quantum Design Physical Properties Measurement System (PPMS Dynacool system) with the vibrating sample magnetometer (VSM) option. The hysteresis loop was recorded at a temperature of 300 K in externally magnetic fields ranging from – 50 kOe to + 50 kOe (in SI unit equal to − 5 to + 5 T). The S-shape of hysteresis loop suggests the system behaves in ferromagnetic (or ferrimagnetic) magnetically ordered regime. The value of saturation magnetization is equal to 20.74 emu/g, the value of remanent magnetization is equal to 1.0 emu/g and the coercive magnetic field is equal to 57.2 Oe. For further details the ZFC-FC measurements should be added.

**
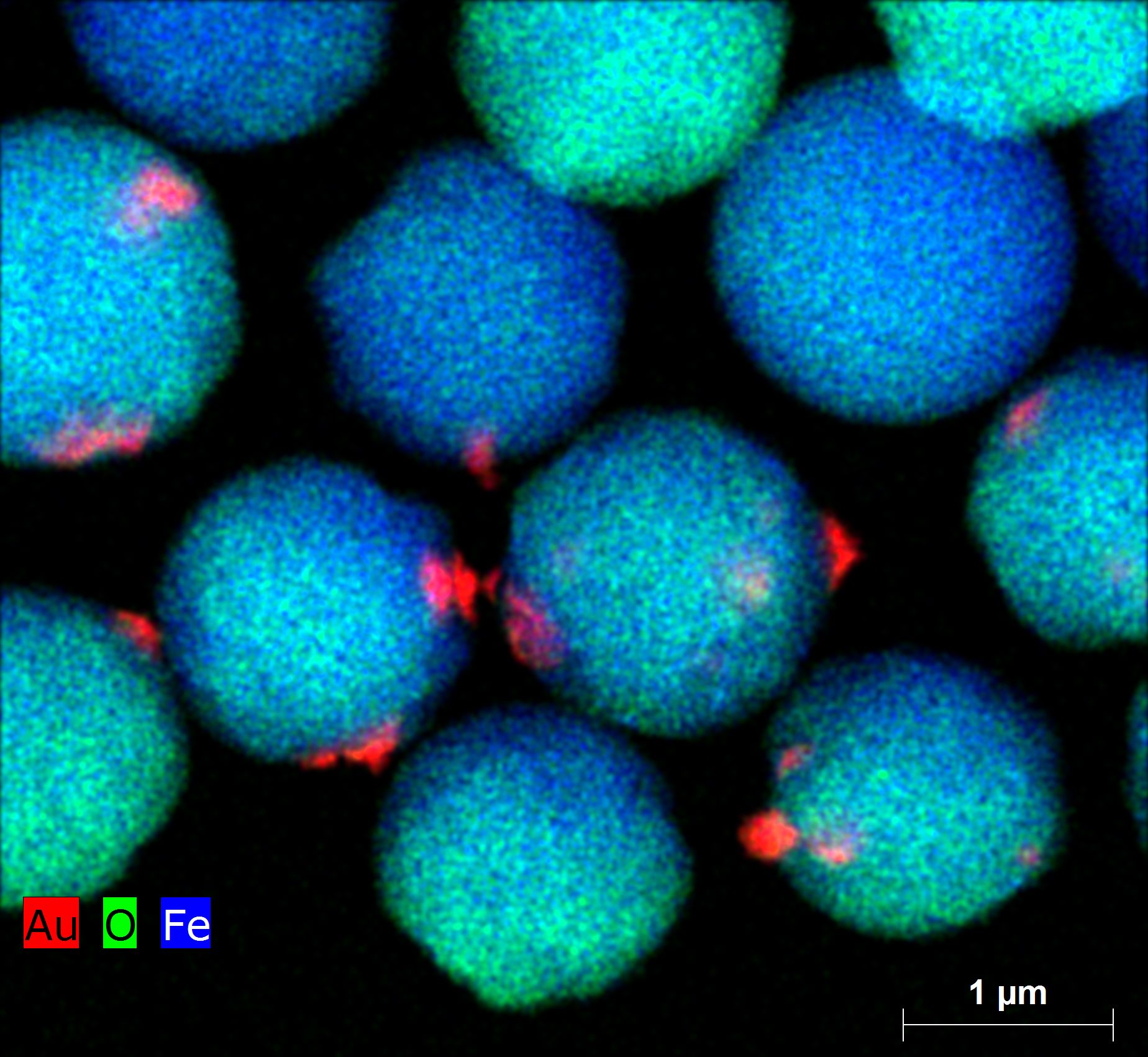
**

**
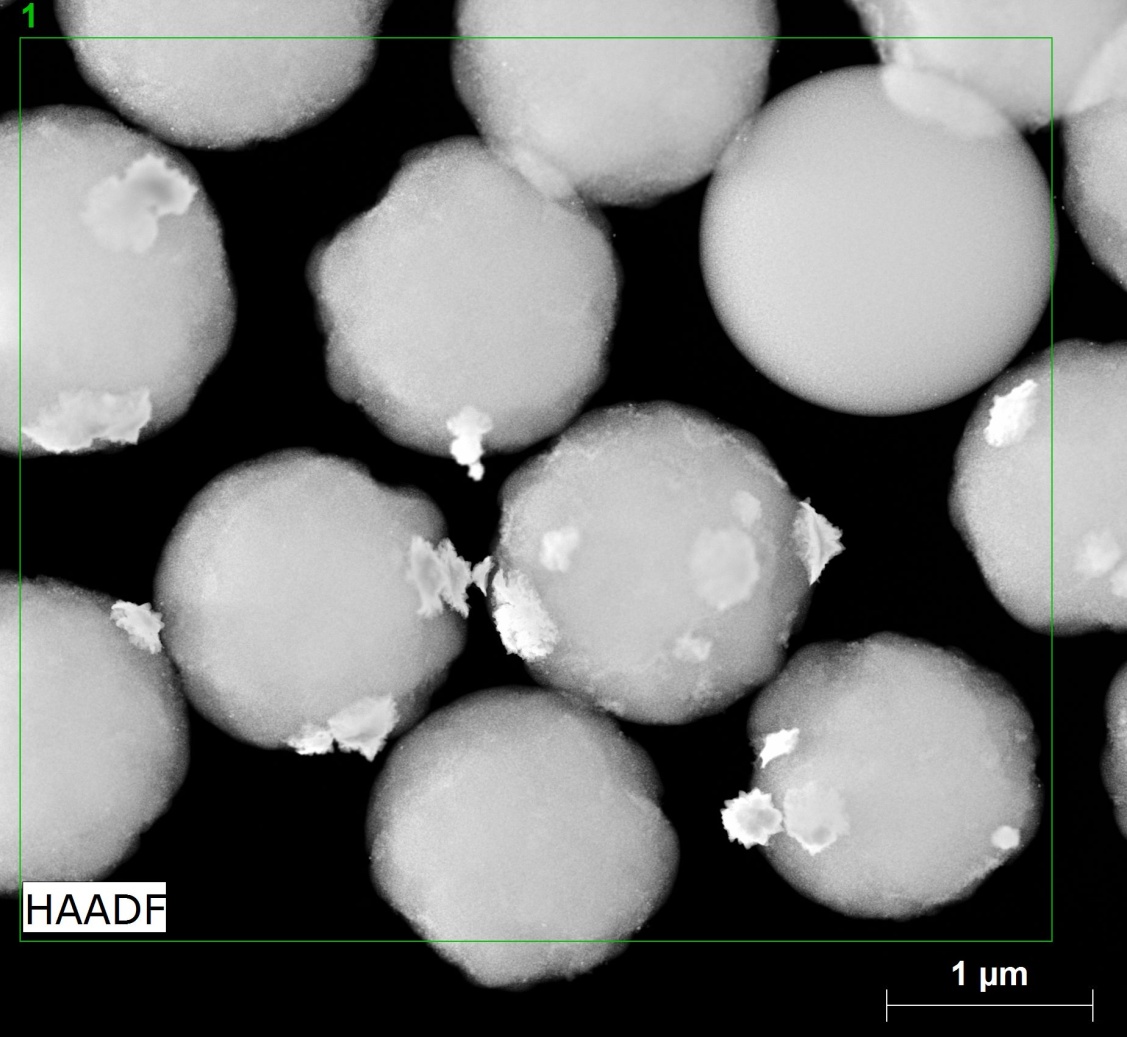
**

**
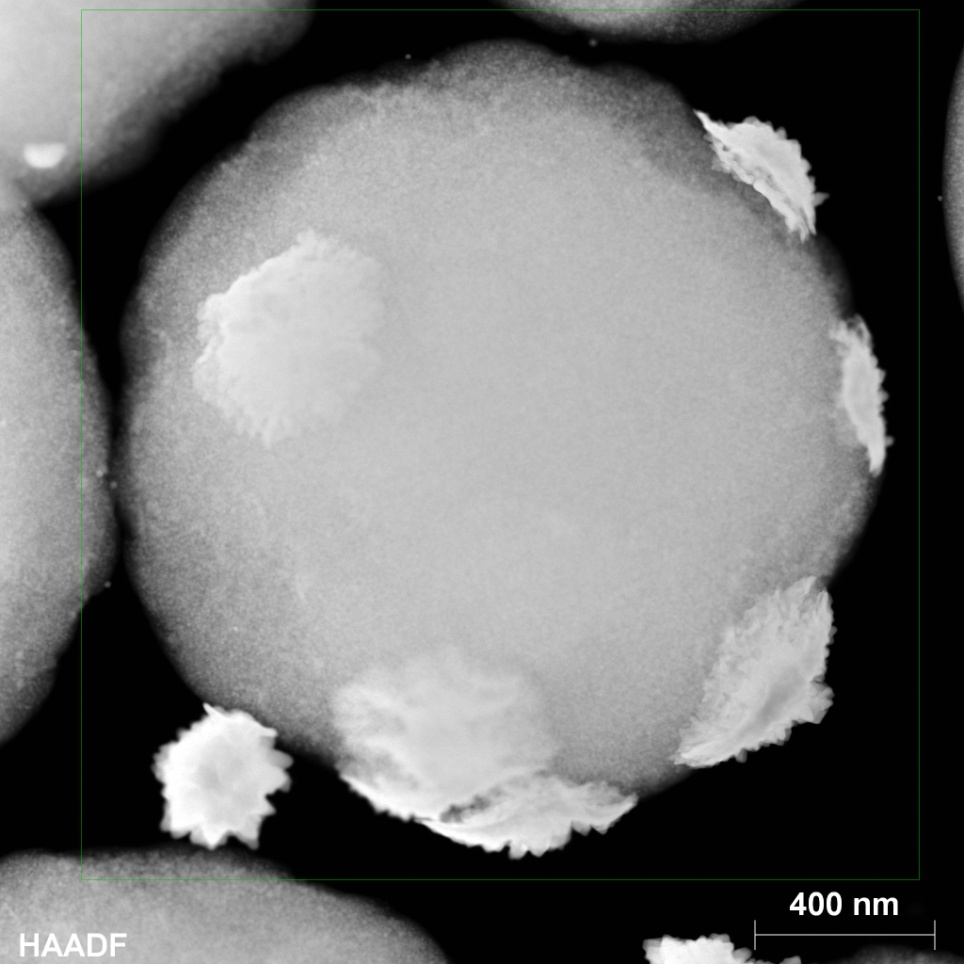
**

**
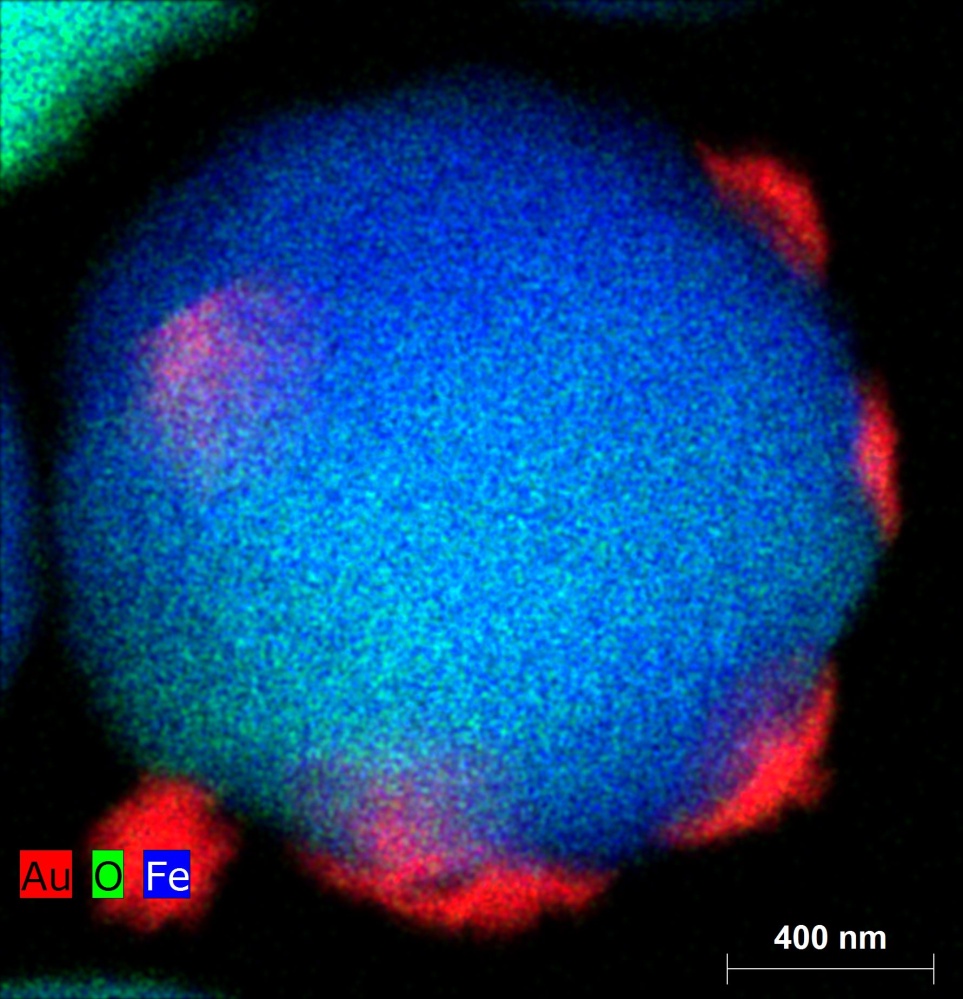
**

**Figures S2:** HR-TEM images and mapping of of elements.
